# Fear, loathing, and support for political violence in the United States: findings from a nationally representative survey

**DOI:** 10.1016/j.lana.2025.101235

**Published:** 2025-09-15

**Authors:** Garen J. Wintemute, Bradley Velasquez, Aaron B. Shev, Elizabeth A. Tomsich, Mona A. Wright, Paul M. Reeping, Sonia L. Robinson, Daniel J. Tancredi, Veronica A. Pear

**Affiliations:** a Centers for Violence Prevention, University of California, Davis, Sacramento, CA, USA; b Department of Emergency Medicine, University of California, Davis, Sacramento, CA, USA; c Department of Pediatrics, University of California, Davis, Sacramento, CA, USA

**Keywords:** Political violence, Firearm violence, Violence and society, Racism, Hostile sexism, Homonegativity, Transphobia, Xenophobia, Antisemitism, Islamophobia, Violent extremism, Civil war

## Abstract

**Background:**

Racism, hostile sexism, homonegativity, transphobia, xenophobia, antisemitism, and Islamophobia increase risk for perpetration of interpersonal violence generally. We quantify their associations, singly and in combination, with support for and willingness to commit political violence in the United States, at a time of increased concern that such violence may become widespread.

**Methods:**

Findings are from Wave 2 of a nationally representative longitudinal survey, conducted May 18-June 8, 2023. Weighted prevalences of the characteristics under study are estimated from levels of agreement with abbreviated versions of previously validated scales, with missing values imputed using multiple imputation by chained equations. Associations with political violence are expressed as adjusted prevalence differences by level of agreement.

**Findings:**

The survey completion rate was 84.2%; there were 9385 respondents. After weighting, 50.7% (95% CI 49.4%–52.1%) of respondents were female; 62.7% (95% CI 61.2%–64.1%) were white, non-Hispanic; the weighted mean (SD) age was 48.5 (25.9) years. Prevalences of strong agreement ranged from 2.9% (95% CI 2.4%–3.4%) for antisemitism to 26.6% (95% CI 25.4%–27.8%) for homonegativity. In all cases, significantly higher percentages of persons in strong agreement than in non-agreement supported and were willing to commit political violence; the largest differences were for Islamophobia and hostile sexism. Less prevalent characteristics tended to have stronger associations with political violence. Associations were larger when characteristics were combined in a single measure.

**Interpretation:**

Racism, hostile sexism, homonegativity, transphobia, xenophobia, antisemitism, and Islamophobia were each strongly associated with support for and willingness to commit political violence in the United States. Such characteristics are resistant to change; prevention efforts should also seek to uncouple them from associated violent behaviors.

**Funding:**

Supported by grants from the 10.13039/100000911Joyce Foundation, 10.13039/100001019California Wellness Foundation, and 10.13039/100014155Heising-Simons Foundation, and by the 10.13039/100020495California Firearm Violence Research Center Violence Prevention Research Program, both at the 10.13039/100007707University of California, Davis.

**Research in context**

**Evidence before this study**

Prior to designing the questionnaire used for this study, the authors considered the literature associating racism, hostile sexism, homonegativity, transphobia, xenophobia, antisemitism, and Islamophobia with risk for committing interpersonal violence and reviewed studies of scales for measuring each of those characteristics. Searches relied on terms in the forms [characteristic + violence] and [characteristic + scale] and proceeded in snowball fashion. Start dates were not specified; end dates were the search dates.

**Added value of this study**

This study adds value to the existing evidence in two ways. It quantifies, for the first time, the associations between seven important forms of fear, hatred, and enmity toward others (considered singly and in combination) and support for and willingness to commit political violence. It also reports the simultaneous population prevalence of those forms of fear, hatred, and enmity toward others in a nationally representative sample, which to our knowledge has not previously been done.

**Implications of all the available evidence**

Each of the characteristics studied is strongly associated with support for and willingness to commit political violence, but all are based on deeply held beliefs and are resistant to change. Prevention strategies may need to focus on uncoupling these characteristics from their associated violent behaviors.

## Introduction

Many forms of hatred, fear, and enmity toward others are widespread and persistent in human populations. When directed at a specific group, the combination of such emotions can be considered akin to a phobia, a term that since the 18th century has comprised fear, horror, strong dislike, and aversion.^1^ Transphobia, xenophobia, and Islamophobia are examples; though they do not incorporate the term, so are homonegativity, racism, sexism, and antisemitism. Collectively, these seven characteristics or phobias can be considered elements of what we term *allophobia*: hatred, fear, and enmity toward an unspecified other. All are strongly and positively associated with perpetration of interpersonal violence in the United States (US).^2^, ^3^, ^4^, ^5^, ^6^, ^7^, ^8^ All have played important roles in the country’s social and political life and will continue to do so.

In recent years, concern for the possibility of widespread political violence has grown in the US.^9^, ^10^, ^11^ Security experts have assessed the threat as arising predominantly from right-wing violent extremism, in which those seven phobias figure prominently.^9^^,^^12^^,^^13^ To our knowledge, associations between those phobias and risk for committing political violence have not been quantified.

In 2022, to gain a deeper understanding of the threat of political violence in the US and to assist prevention efforts, we initiated a nationally representative annual longitudinal survey to examine support for and willingness to commit political violence and to identify sources of variation in those measures.^14^ Racism was strongly associated with support for political violence in that first wave of the survey; 51.1% of respondents who strongly agreed with statements of racist beliefs, but only 21.6% of those who did not agree, considered physical violence to be usually or always justified to advance at least 1 of 17 specified political objectives.^15^ Those who strongly agreed also more frequently predicted that they would be armed with a firearm in a future situation where they considered political violence to be justified (strong agreement, 11.1%; non-agreement, 3.9%).^15^

Given those concerning findings, we expanded this line of inquiry in 2023’s Wave 2 of the survey, measuring endorsement of the seven phobias mentioned above using abbreviated versions of previously validated scales.^16^, ^17^, ^18^, ^19^, ^20^, ^21^ We report here their prevalences and cross-sectional estimates of their associations, singly and in combination as allophobia, with support for and willingness to commit political violence. These findings may be of significant population-level benefit, in that they will contribute to our understanding of and ability to prevent political violence.

## Methods

Wave 2 of the survey was designed by the authors and administered online in English and Spanish from May 18 to June 8, 2023, by the survey research firm Ipsos.

### Ethics approval

The study was reviewed by the University of California Davis Institutional Review Board (IRB Protocol 1,871,725, March 30, 2023; Exempt: Category 2—survey research meeting additional criteria regarding risk to participants). Written or verbal consent was not required by the IRB; before accessing the questionnaire, participants were provided consent language that concluded, “[by] continuing, you are agreeing to participate in this study.” All authors participated in the analysis of the data and/or the interpretation of the findings. The study is reported following American Association for Public Opinion Research guidelines.^22^

### Participants

Survey participants were drawn from the Ipsos KnowledgePanel. To establish a nationally representative panel, KnowledgePanel members are recruited on an ongoing basis through address-based probability sampling using data from the US Postal Service’s Delivery Sequence File.^23^^,^^24^ Recruitment into KnowledgePanel involves repeated contact attempts by mail and telephone. Recruited adults in households without internet access are provided a web-enabled device and free internet service. A modest incentive program seeks to encourage participation and promote participants’ retention in KnowledgePanel over time.^23^^,^^24^

A probability-proportional-to-size procedure was used to select a study-specific nationally representative sample of KnowledgePanel members for Wave 1. All panel members who were aged 18 years and older in 2022 were eligible for selection. Invitations were sent by e-mail; automatic reminders were delivered to non-respondents by e-mail and telephone beginning three days later.^23^^,^^24^

The Wave 1 survey was conducted May 13 to June 2, 2022.^14^ It included a main sample, which had a completion rate of 53%, and oversamples of firearm owners, transgender people, combat veterans, and California residents to support preplanned supplemental analyses. Main sample respondents were older than nonrespondents and more frequently white, non-Hispanic; were more often married; had higher education and income; and were less likely to be working.^14^ Altogether, Wave 1 included 12,947 respondents. Invitations to participate in Wave 2 were sent to the 11,140 Wave 1 respondents (86.0%) who remained active members of KnowledgePanel on Wave 2’s launch date (The remaining 1807 Wave 1 respondents had left the cohort through normal attrition.)

A final Wave 2 survey weight variable provided by Ipsos adjusted for the initial probability of selection into KnowledgePanel and for survey-specific nonresponse and over- or under-coverage using design weights with post-stratification raking ratio adjustments with benchmarks obtained from the 2021 March supplement of the Current Population Survey.^23^^,^^24^

### Measures

Sociodemographic data were collected by Ipsos from profiles created and maintained by KnowledgePanel members. The seven phobias we included were chosen for their established associations with interpersonal violence.^2^, ^3^, ^4^, ^5^, ^6^, ^7^, ^8^ Survey items that supplied data on phobias were obtained in all but one case from validated scales (Table 1).^16^, ^17^, ^18^, ^19^, ^20^, ^21^ These scales were selected based on recency of development, extent of validation, and applicability to estimating associations between the phobias they measured and political violence (We chose to measure homonegativity rather than homophobia as validated scales for the latter were often decades old and obviously dated.) Questionnaire length limitations precluded use of complete scales; we included items based on their direct relevance to violence. An abbreviated validated scale for racism was included in the questionnaire, but the selected items did not have an acceptable Cronbach’s α (a measure of internal consistency among items in a scale). Analyses for racism relied instead on four items regarding racism that had a Cronbach’s α of 0.81 in our 2022 survey^14^ and were included in the 2023 questionnaire.

**Table 1:** Construction and prevalence of individual phobias and allophobia (n = 9385).

Phobia	Cronbach’s α (95% CI)	Prevalence by level of agreement	
		Unweighted n	Weighted % (95% CI)
**Homonegativity**	0.900 (0.897,0.903)		
Strong agreement		2898	26.6 (25.4,27.8)
Moderate agreement		2353	25.5 (24.2,26.7)
Weak agreement		2860	33.4 (32.0,34.7)
Non-agreement		1275	14.5 (13.6,15.5)
**Racism**	0.781 (0.773,0.788)		
Strong agreement		2344	19.5 (18.5,20.6)
Moderate agreement		3181	35.2 (33.8,36.6)
Weak agreement		2197	25.4 (24.2,26.7)
Non-agreement		1663	19.8 (18.7,20.9)
**Transphobia**	0.829 (0.824,0.834)		
Strong agreement		1710	16.9 (15.8,17.9)
Moderate agreement		3036	31.3 (29.9,32.6)
Weak agreement		3544	37.7 (36.4,39.1)
Non-agreement		1096	14.2 (13.1,15.2)
**Xenophobia**	0.816 (0.809,0.822)		
Strong agreement		1073	9.8 (9.0,10.6)
Moderate agreement		2042	20.8 (19.6,21.9)
Weak agreement		4102	43.7 (42.3,45.1)
Non-agreement		2168	25.7 (24.5,27.0)
**Hostile sexism**	0.866 (0.861,0.872)		
Strong agreement		623	7.7 (6.9,8.5)
Moderate agreement		1509	17.2 (16.1,18.3)
Weak agreement		3923	38.4 (37.1,39.8)
Non-agreement		3331	36.7 (35.4,38.1)
**Islamophobia**	0.846 (0.838,0.854)		
Strong agreement		489	5.0 (4.4,5.7)
Moderate agreement		1184	12.8 (11.8,13.8)
Weak agreement		3081	29.4 (28.1,30.6)
Non-agreement		4632	52.8 (51.4,54.2)
**Antisemitism**	0.755 (0.743,0.766)		
Strong agreement		226	2.9 (2.4,3.4)
Moderate agreement		1130	17.4 (16.2,18.6)
Weak agreement		2709	28.7 (27.4,30.0)
Non-agreement		5319	51.0 (49.6,52.4)
**Allophobia**	Not applicable		
Strong agreement		491	4.6 (4.0,5.1)
Moderate agreement		3421	34.7 (33.4,36.1)
Weak agreement		5226	57.6 (56.2,59.0)
Non-agreement		248	3.1 (2.6,3.5)

Phobias are presented in order of prevalence of strong agreement.

Response options for individual items were “do not agree,” “somewhat agree,” “strongly agree,” and “very strongly agree.” Details of categorization of respondents by level of agreement with individual phobias and with allophobia (a combined measure) are provided in the methods section.

Test of individual items included in scales (R following an item denotes reverse coding).

Homonegativity: Celebrations such as “Gay Pride Day” are ridiculous, because they assume that an individual’s sexual orientation should constitute a source of pride. Gay men and lesbian women should stop shoving their lifestyle down other people’s throats. Many gay men and lesbian women use their sexual orientation so that they can obtain special rights and privileges. Gay men and lesbian women who are “out of the closet” should be admired for their courage (R). In today’s tough economic times, Americans’ tax dollars shouldn’t be used to support gay and lesbian organizations. Gay men and lesbian women should stop complaining about the way they are treated in society, and simply get on with their lives.

Racism: White people benefit from advantages in society that Black people do not have (R). Discrimination against whites is as big a problem as discrimination against Blacks and other minorities. A group of people in this country is trying to replace native-born Americans with immigrants and people of color who share their political views. Having more Black Americans, Latinos, and Asian Americans is good for the country (R).

Transphobia: I think there is something wrong with a person who says that they are neither a man nor a woman. I would be upset, if someone I’d known a long time revealed to me that they used to be another gender. I avoid people on the street whose gender is unclear to me. When I meet someone, it is important for me to be able to identify them as a man or a woman. I believe that the male/female dichotomy is natural. I believe that a person can never change their gender.

Xenophobia: Interacting with immigrants makes me uneasy. Immigrants cause an increase in crime. I enjoy interacting with immigrants (R). I am afraid that our own culture will be lost with an increase in immigration. I am afraid that in case of political tension, immigrants will be loyal to their country of origin.

Hostile sexism: Women seek to gain power by getting control over men. Women exaggerate problems they have at work. Once a woman gets a man to commit to her, she usually tries to put him on a tight leash. When women lose to men in a fair competition, they typically complain about being discriminated against. Many women get a kick out of teasing men by seeming sexually available and then refusing male advances. Feminists are making unreasonable demands of men.

Islamophobia: Most Muslims living in the United States are more prone to violence than other people. Most Muslims living in the United States discriminate against women. Most Muslims living in the United States are hostile to the United States. Most Muslims living in the United States are less civilized than other people.

Antisemitism: Jewish people can be trusted just as much as other Americans in business (R). Jewish people are just as loyal to the United States as other Americans (R). Compared to other groups, Jewish people have too much power in the media. Jewish people talk about the Holocaust just to further their political agenda. Jewish people chase money more than other people do.

Our outcome measures concerned political violence. Violence was represented in the questionnaire by “force or violence,” defined as “physical force strong enough that it could cause pain or injury to a person.” “Force or violence to advance an important political objective that you support” was used to denote political violence.

Respondents were asked about the extent to which they considered political violence to be justified “in general” and then about justification for its use to advance 19 specified political objectives (examples: “to return Donald Trump to the presidency this year,” “to stop police violence”).

Respondents who considered violence at least sometimes justified to advance at least 1 of these 19 objectives were asked about their personal willingness to commit political violence: for four types of violence (to “damage property,” “threaten or intimidate a person,” “injure a person,” “kill a person”) and against 11 target populations (examples: “an elected federal or state government official,” “a police officer,” “a person who does not share your religion”).

All respondents were asked about the likelihood of their future use of firearms in a situation where they considered political violence justified (examples: “I will be armed with a gun”; “I will shoot someone with a gun”).

The text of all items included in this analysis is in the Supplement.

### Implementation

Ipsos translated the questionnaire into Spanish, and interpreting services staff at UC Davis reviewed the translation. Thirty-three KnowledgePanel members participated in a pretest of the English language version, administered May 5–9, 2023.

Respondents were randomized 1:1 to receive response options in order from negative to positive valence (e.g., from ‘do not agree’ to ‘strongly agree’) or the reverse throughout the questionnaire. When an item presented multiple statements for respondents to consider, the presentation order was randomized unless ordering was necessary.

We employed unipolar response arrays without neutral midpoints (example: do not agree, somewhat agree, strongly agree, very strongly agree), given evidence that midpoints facilitate satisficing: selection of “a minimally acceptable response as soon as it is found.”^25^ Satisficing is particularly common when respondents are uncomfortable with the topics of the survey or under social desirability pressures, and both conditions apply here. We also focused our analyses on expressions of support for and willingness to commit political violence above the “somewhat” or “sometimes” level to minimize the impact of satisficing on the results.

### Statistical analysis

All analyses were conducted in R version 4.3.0 (R Foundation).

Approximately 4.3% of respondents were missing more than half of the items for at least one individual phobia, and approximately 7.1% of respondents were missing at least one item. These missing values were imputed using Multiple Imputation by Chained Equations (MICE) to create five imputed data sets after five iterations of the chain.

Individual item responses for the seven phobias were then coded ordinally (e.g., do not agree = 0, somewhat agree = 1, strongly agree = 2, very strongly agree = 3) and summed for each respondent for each phobia. Sums were normalized to a range from 0 to 1, with 0 and 1 representing the minimum and maximum theoretically possible scores. Respondents’ phobia scores were then categorized according to their position on that range (e.g., strong agreement, phobia score >0.66… and ≤1; moderate agreement, phobia score >0.33… and ≤0.66; weak agreement, phobia score >0 and ≤ 0.33…; non-agreement, phobia score = 0).

A respondent’s allophobia score was defined as the sum of the individual phobia scores. Each phobia’s proportional contribution to the allophobia score was computed by dividing the individual phobia score by the allophobia score.

Cronbach’s alphas and their 95% confidence intervals (CIs) were calculated using the estimates and standard errors pooled across the imputed data sets according to Rubin’s rules.^26^ Standard errors for Cronbach’s alpha in each imputed data set were computed using a basic bootstrap method with 1000 resamples.

To assess associations among phobias, we generated a correlation heat map based on normalized individual phobia scores, using the *ggplot2* package to produce weighted Pearson correlation coefficients.

To generate prevalence estimates, we calculated weighted percentages and 95% CIs using the *survey* and *srvyr* packages. Weighted prevalences and adjusted prevalence differences (aPDs, which are percentage point (pp) differences) were pooled from the multiple imputation results. We employed linear regression models to compute aPDs and their 95% CIs, defining outcomes dichotomously and employing robust standard errors to correct for design effects and heteroskedasticity in binary outcomes. We had previously found^14^ that sociodemographic characteristics were associated with our political violence outcome measures. The final regression model included those characteristics—age, race and ethnicity, gender, education, income, Census division, and rurality—and produced the best fit statistics (see Supplement).

P values were corrected for multiple comparisons by controlling the false discovery rate (FDR) using the Benjamini-Hochberg method^27^ and are reported as q values.^28^ Q values represent the probability that the given difference would be a false discovery: the expected proportion of “false positives” that would be seen among the collection of all differences whose q values were at or below the given q value.

In a sensitivity analysis, we assessed bounds on potential bias due to our imputation by inserting the minimum and maximum possible values for missing responses and calculating the percent change in respondent scores across imputations.

### Role of the funding sources

The study sponsors played no role in the study design; in the collection, analysis, and interpretation of data; in the writing of the report; or in the decision to submit the paper for publication.

## Results

Of 11,140 panel members invited to participate, 9385 completed the survey, yielding an 84.2% completion rate. The median survey completion time was 25 min (interquartile range, 18.6 min). Item non-response ranged from 0.4% to 5.4%; seven items had non-response percentages above 3.0% (see Supplement).

After weighting, half of the respondents (50.7%, 95% CI 49.4%–52.1%) were female; 62.7% (95% CI 61.2%–64.1%) were white, non-Hispanic (Supplementary Table S1). The weighted mean (SD) respondent age was 48.5 (25.9) years. Compared with nonrespondents, respondents were older; more frequently male, non-Hispanic white, and married; and less frequently working full-time (Supplementary Table S2).

Cronbach’s α values for the seven scales ranged from 0.755 (95% CI 0.743–0.766) for antisemitism to 0.900 (95% CI 0.897–0.903) for homonegativity (Table 1). The prevalence of strong agreement ranged from 2.9% (95% CI 2.4%–3.4%) for antisemitism to 26.6% (95% CI 25.4%–27.8%) for homonegativity (Table 1). For allophobia, the prevalence of strong agreement was 4.6% (95% CI 4.0%–5.1%). Supplementary Table S3 contains detailed results for all items used to construct the seven scales.

**Fig. 1:**
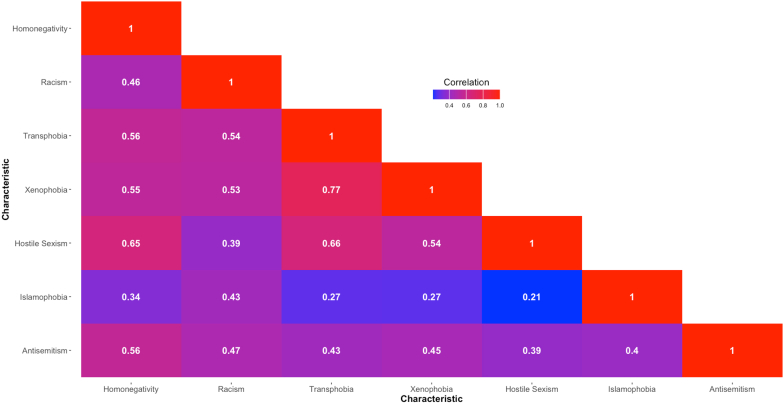
Correlation heat map for seven phobias (n = 9385). Values are weighted Pearson correlation coefficients. P values for all correlations are <0.001.

Phobias were moderately to highly correlated (Fig. 1); p values for all correlations were <0.001. Correlations were highest between xenophobia and transphobia (r = 0.77), hostile sexism and transphobia (r = 0.66), and hostile sexism and homonegativity (r = 0.65). Islamophobia had the lowest correlations with other phobias (r = 0.21–0.43).

**Fig. 2:**
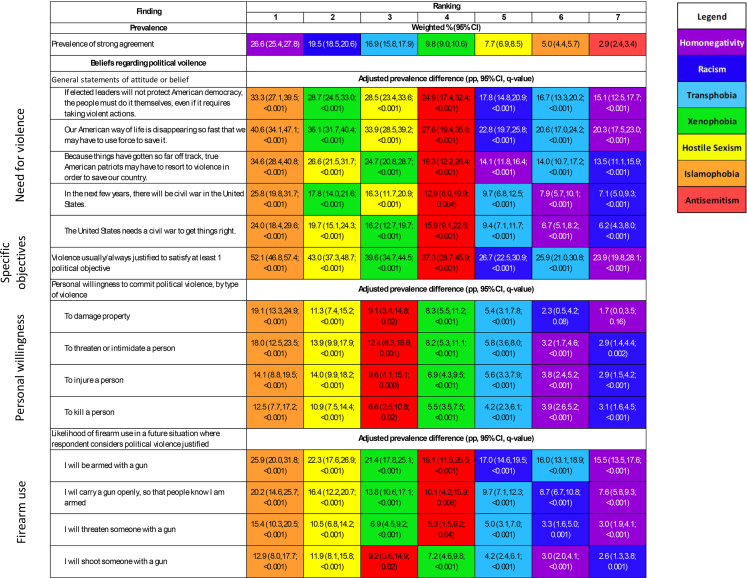
Ranked prevalences, and ranked adjusted prevalence differences for political violence measures, for each of seven phobias (n = 9385). Prevalence: Phobias are ranked by the prevalence of strong agreement. See the methods section or notes to Table 1 for details of agreement categorization. Outcome measures: Phobias are ranked by the adjusted prevalence difference for each outcome measure between respondents classified as in strong agreement and those classified as in non-agreement with the phobia. Prevalence differences are percentage point (pp) differences. They are weighted and adjusted for age, race and ethnicity, gender, education, income, Census division, and rurality. Response options for which these differences were calculated are as follows: for need for violence to effect social change and for civil war, strongly or very strongly agree; for violence to advance specific political objectives, “force or violence to advance an important political objective” was usually or always justified to advance at least 1 of 19 such objectives; for personal willingness to commit political violence, very or completely willing; for expectations of firearm use, very or extremely likely. Q values represent the probability that the given difference would be a false discovery; they represent the expected proportion of “false positives” that would be seen among the collection of all differences whose q values were at or below the given q value.

There were significant associations between level of agreement with each phobia and measures of support for political violence, including a perceived need for civil war; with personal willingness to commit political violence; and with the use of firearms in political violence. These findings are summarized in Fig. 2, which presents aPDs between respondents in strong agreement and those in non-agreement for all seven phobias, and are presented in detail for each phobia individually in Supplementary Tables S4–S10.

For Islamophobia, for example (Fig. 2, Supplementary Table S9), aPDs between those in strong agreement and those in non-agreement ranged from 12.5 pp (95% CI 7.7 pp, 17.2 pp; p < 0.001) for willingness to kill another person to advance a political objective to 52.1 pp (95% CI 46.8 pp, 57.4 pp; p < 0.001) for the belief that violence was usually or always justified to satisfy at least one political objective.

There were only two instances of non-association. Homonegativity (Fig. 2, Supplementary Table S4) and racism (Fig. 2, Supplementary Table S5) were not associated with willingness to damage property to advance a political objective (homonegativity: aPD 1.7 pp, 95% CI 0.0 pp, 3.5 pp; p = 0.16) (racism: aPD 2.3 pp, 95% CI 0.5 pp, 4.2 pp; p = 0.08).

Also as shown in Fig. 2, there was an inverse relationship between prevalences of strong agreement with phobias (displayed at the top of the figure) and the magnitude of phobias’ associations with political violence measures; less prevalent phobias were more strongly associated with political violence. APDs between respondents in strong agreement and those in non-agreement were consistently largest for Islamophobia, hostile sexism, xenophobia, and antisemitism.

For any single phobia, associations tended to be larger for general statements of attitude or belief regarding political violence than for measures of personal willingness to commit political violence and for likelihood of firearm use (Fig. 2; Supplementary Tables S4–S10).

**Fig. 3:**
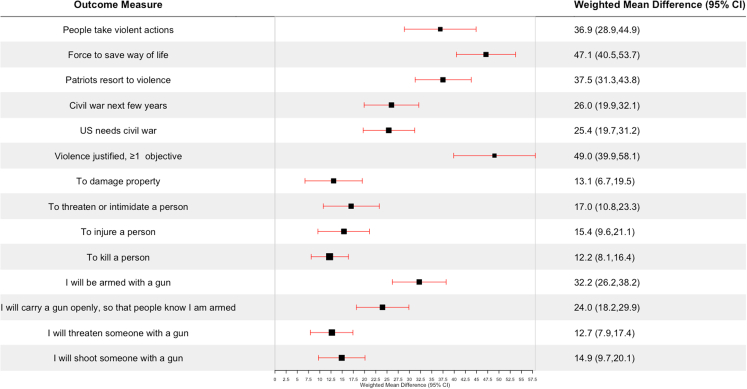
Allophobia and political violence (n = 9385). The figure presents the adjusted weighted percentage point difference between respondents classified as in strong agreement and those classified as in non-agreement with allophobia, which combines items from scales for all seven individual phobias. The full text of the items summarized in the outcome measure column, and the response on which the percentage point difference is computed, are as follows: People take violent actions: If elected leaders will not protect American democracy, the people must do it themselves, even if it requires taking violent actions (Strongly/very strongly agree). Force to save way of life: Our American way of life is disappearing so fast that we may have to use force to save it (Strongly/very strongly agree). Patriots resort to violence: Because things have gotten so far off track, true American patriots may have to resort to violence in order to save our country (Strongly/very strongly agree). Civil war next few years: In the next few years, there will be civil war in the United States (Strongly/very strongly agree). US needs civil war: The United States needs a civil war to set things right (Strongly/very strongly agree). Violence justified, ≥1 objective: “force or violence to advance an important political objective” usually/always justified to advance at least 1 of 19 such objectives. Personal willingness measures: In a situation where you think force or violence is justified to advance an important political objective, how willing would you personally be to use force or violence in each of these ways? The full text of these items is in the figure (Very/completely willing). Future use of firearm measures: Thinking now about the future and all the changes it might bring, how likely is it that you will use a gun in any of the following ways in the next few years—in a situation where you think force or violence is justified to advance an important political objective? The full text of these items is in the figure (Very/extremely likely).

Findings for allophobia, the combined measure, are summarized in Fig. 3 and presented in detail in Supplementary Table S11. Most associations between strong agreement and political violence measures were larger for allophobia than for any individual phobia; examples are “the United States needs a civil war to set things right” (allophobia: aPD 25.4 pp, 95% CI 19.7 pp, 31.2 pp; p < 0.001) and “I will shoot someone with a gun” (allophobia: aPD 14.9 pp, 95% CI 9.7 pp, 20.1 pp; p < 0.001).

### Sensitivity analysis

There were small differences between MICE and minimum-value imputation (average percent change −3.94% (0.275 SE)). The difference between MICE and maximum-value imputation was larger (average percent change 17.14% (2.425 SE)). This was expected, as maximum-value responses were uncommon in the observed data.

## Discussion

This large, nationally representative survey of adults in the United States found concerningly high prevalences of seven forms of irrational hatred, fear, and enmity toward others that can usefully be described as—and three of which are routinely named as—phobias. The principal aim of this study was to assess their associations with political violence. Such associations exist in all seven cases and are comparable in magnitude to those we have found for MAGA Republicans,^29^ supporters of extreme right-wing political organizations and movements such as the Proud Boys and QAnon,^15^ and firearm owners who own assault-type rifles or regularly carry loaded firearms in public.^30^

There is a striking inverse association between the seven phobias’ prevalence and the magnitude of their association with political violence. Perhaps strong endorsement of an uncommon phobia represents a more extreme position than does strong endorsement of a common phobia; extremism is strongly associated with violence.^31^

There is a moderate to high degree of correlation in the prevalence of these phobias, as other more limited explorations^32^ have found and as was observed by developers of some of the scales used here.^16^^,^^17^^,^^20^ Such co-occurrence is concordant with social dominance theory, which views attributes such as the phobias we assessed here as “special cases of a more general tendency for humans to form and maintain group-based hierarchy.”^33^

The finding of stronger associations for allophobia than for individual phobias has several potential explanations. For example, individual phobias may interact positively in their association with political violence. Alternatively, given extremism’s association with violence, it may simply be that allophobia (a combined measure) is a better proxy for extremism than individual phobias are.

What are the implications of these findings? Persons who endorse racism, hostile sexism, homonegativity, transphobia, xenophobia, antisemitism, and Islamophobia should be viewed as being at increased risk for committing political violence generally, not just violence directed at populations suggested by the phobia(s) they endorse. Political violence prevention efforts could focus on such individuals, as they should on members of other high-risk groups that have been identified previously.^14^^,^^15^^,^^29^^,^^30^

Because phobias are deeply held beliefs that resist change,^34^ interventions designed to uncouple such beliefs from participation in political violence may prove more efficacious in the near term. They should be seen as complementary to continuing efforts to reduce the prevalence of phobic attitudes and beliefs.

Suasion might prove to be a successful uncoupling strategy.^35^^,^^36^ For example, our survey has found that among those who consider it very or extremely likely that they would serve as combatants if civil war broke out in the US, as many as 45% would abandon that position if urged to do so by family members and others.^37^ Other approaches will be needed for those not susceptible to suasion, including traditional threat assessment and law enforcement investigative efforts. Assessments related to firearm access in the US, such as for permits to purchase firearms or carry concealed firearms and for extreme risk protection orders,^38^ could include records of an individual’s acts or threats of violence against marginalized groups. Such a policy has recently been enacted for extreme risk protection orders in California.^39^

### Limitations

The findings are cross-sectional. Weighting notwithstanding, they may be affected by sampling error, non-response bias arising from differences between respondents and nonrespondents, undercoverage bias arising from USPS address-based sampling, selection bias at the time of recruitment into KnowledgePanel, social desirability bias given the topics addressed in the survey, and other threats to validity. Potential effects of these sources of error are mixed. For example, nonrespondents are younger and more frequently female that respondents. The former difference suggests that our findings might underestimate willingness to commit political violence, the high response rate and weighting notwithstanding; the latter difference suggests the opposite.^14^

Other scales exist for measuring the phobias we studied and might have produced different findings. Measuring homophobia rather than homonegativity, for example, might have produced a lower prevalence and a larger association with political violence.^40^ Our survey was conducted in mid-2023. Since then, events in the Middle East and in the US have likely affected prevalences of Islamophobia and antisemitism, and continued controversy over policies related to reproductive rights, sexual orientation, and gender identity in the US has likely affected prevalences of hostile sexism, homonegativity and transphobia. A few outcomes are uncommon and estimates are potentially unstable, particularly for respondents classified as in non-agreement with the seven phobias we studied.

### Conclusion

Findings from this large, nationally representative survey suggest that prevalences of racism, hostile sexism, homonegativity, transphobia, xenophobia, antisemitism, and Islamophobia are concerningly high in the US and that these phobias, individually and in combination, are strongly associated with support for political violence. Such findings should be used to sharpen the focus of political violence prevention efforts.

## Contributors

GW: conception and design; acquisition, analysis, and interpretation of data; drafting of manuscript.

BV: conception and design; acquisition, analysis, and interpretation of data; substantive revision of manuscript.

AS: conception and design; acquisition, analysis, and interpretation of data; substantive revision of manuscript.

ET: conception and design; acquisition, analysis, and interpretation of data; substantive revision of manuscript.

MW: conception and design; acquisition, analysis, and interpretation of data; substantive revision of manuscript.

PR: conception and design; acquisition, analysis, and interpretation of data; substantive revision of manuscript.

SR: conception and design; acquisition, analysis, and interpretation of data; substantive revision of manuscript.

DT: conception and design; acquisition, analysis, and interpretation of data; substantive revision of manuscript.

VP: conception and design; acquisition, analysis, and interpretation of data; substantive revision of manuscript.

All authors had full access to the data and read and approved the final manuscript.

## Data sharing statement

The datasets generated and/or analyzed during the current study will be made available to qualified researchers subject to the terms of a data use agreement.

## Artificial intelligence use statement

No artificial intelligence tools were used in the research or manuscript preparation.

## Declaration of interests

The authors have no competing interests to report.
