# Supplementary material for: Fear, loathing, and support for political violence in the United States: findings from a nationally representative survey
Source: Lancet Reg Health Am. 2025 Sep 15;51:101235. doi: 10.1016/j.lana.2025.101235 (PMC12464958; doi:10.1016/j.lana.2025.101235)
Supplement: Supplementary Tables S1–S11 [file mmc1.docx]

**Supplement:** **Fear, Loathing, and Support for Political Violence in the United States: Findings from a Nationally Representative Survey**

Garen J. Wintemute, MD; Bradley Velasquez, MS; Elizabeth A. Tomsich, PhD; Paul M. Reeping, PhD; Sonia L. Robinson, PhD; Dan Tancredi, PhD; Veronica A. Pear, PhD

This supplement has been provided by the authors to give readers additional information about the work.

| **Page** | **Title** |
| --- | --- |
| 2 | Questions that supplied data for this study |
| 7 | Additional methods text |
| 8 | Additional results text |
| 9 | Table S1. Sociodemographic characteristics of respondents (weighted) |
| 11 | Table S2. Sociodemographic characteristics of respondents and non-respondents in the 2022 and 2023 surveys (unweighted) |
| 13 | Table S3. Prevalence of agreement with individual items used to construct phobia scales |
| 16 | Table S4. Level of agreement with statements of homonegativity and beliefs regarding political violence |
| 18 | Table S5. Level of agreement with statements of racism and beliefs regarding political violence |
| 20 | Table S6. Level of agreement with statements of transphobia and beliefs regarding political violence |
| 22 | Table S7. Level of agreement with statements of xenophobia and beliefs regarding political violence |
| 24 | Table S8. Level of agreement with statements of hostile sexism and beliefs regarding political violence |
| 26 | Table S9. Level of agreement with statements of Islamophobia and beliefs regarding political violence |
| 28 | Table S10. Level of agreement with statements of antisemitism and beliefs regarding political violence |
| 30 | Table S11. Level of agreement with allophobia and beliefs regarding political violence |
| 32 | References |

**QUESTIONS THAT SUPPLIED DATA FOR THIS STUDY**

Response options are presented here in order from negative to positive (e.g., “not important” to “extremely important”). Respondents were randomized 1:1 to receive responses in that order or the reverse.

In the list below, questions or items that were repeated or adapted from prior surveys by other investigators contain citations to those surveys.

**Homonegativity**

**Q:** How much do you agree or disagree with each of the following statements about gay men and lesbian women in the United States today?^1^

a. Celebrations such as “Gay Pride Day” are ridiculous, because they assume that an individual’s sexual orientation should constitute a source of pride.

b. Gay men and lesbian women should stop shoving their lifestyle down other people’s throats.

c. Many gay men and lesbian women use their sexual orientation so that they can obtain special rights and privileges.

d. Gay men and lesbian women who are “out of the closet” should be admired for their courage.

e. In today's tough economic times, Americans' tax dollars shouldn't be used to support gay and lesbian organizations.

f. Gay men and lesbian women should stop complaining about the way they are treated in society, and simply get on with their lives.

1. Do not agree

2. Somewhat agree

3. Strongly agree

4. Very strongly agree

**Racism**

**Q:** How much do you agree or disagree with each of the following statements about people in the United States today?

a. White people benefit from advantages in society that Black people do not have.^2^ (Reverse coded.)

b. Discrimination against whites is as big a problem as discrimination against Blacks and other minorities. ^3^

c. A group of people in this country is trying to replace native-born Americans with immigrants and people of color who share their political views.

d. Having more Black Americans, Latinos, and Asian Americans is good for the country.^4^ (Reverse coded.)

1. Do not agree

2. Somewhat agree

3. Strongly agree

4. Very strongly agree

**Transphobia**

**Q:** How much do you agree or disagree with each of the following statements about gender in the United States today?^5^

a. I think there is something wrong with a person who says that they are neither a man nor a woman.

b. I would be upset, if someone I’d known a long time revealed to me that they used to be another gender.

c. I avoid people on the street whose gender is unclear to me.

d. When I meet someone, it is important for me to be able to identify them as a man or a woman.

e. I believe that the male/female dichotomy is natural.

f. I believe that a person can never change their gender.

1. Do not agree

2. Somewhat agree

3. Strongly agree

4. Very strongly agree

**Xenophobia**

**Q:** How much do you agree or disagree with each of the following statements about people who have immigrated to the United States?^6^

a. Interacting with immigrants makes me uneasy.

b. Immigrants cause an increase in crime.

c. I enjoy interacting with immigrants.

d. I am afraid that our own culture will be lost with an increase in immigration.

e. I am afraid that in case of political tension, immigrants will be loyal to their country of origin.

1. Do not agree

2. Somewhat agree

3. Strongly agree

4. Very strongly agree

**Hostile Sexism**

**Q:** How much do you agree or disagree with each of the following statements about women in the United States today?^7^

a. Women seek to gain power by getting control over men.

b. Women exaggerate problems they have at work.

c. Once a woman gets a man to commit to her, she usually tries to put him on a tight leash.

d. When women lose to men in a fair competition, they typically complain about being discriminated against.

e. Many women get a kick out of teasing men by seeming sexually available and then refusing male advances.

f. Feminists are making unreasonable demands of men.

1. Do not agree

2. Somewhat agree

3. Strongly agree

4. Very strongly agree

**Islamophobia**

**Q:** How much do you agree or disagree with each of the following statements about people’s religious beliefs in the United States today?^8^

a. Most Muslims living in the United States are more prone to violence than other people.

b. Most Muslims living in the United States discriminate against women.

c. Most Muslims living in the United States are hostile to the United States.

d. Most Muslims living in the United States are less civilized than other people.

1. Do not agree

2. Somewhat agree

3. Strongly agree

4. Very strongly agree

**Antisemitism**

**Q:** How much do you agree or disagree with each of the following statements about people’s religious beliefs in the United States today?^9^

a. Jewish people can be trusted just as much as other Americans in business.

b. Jewish people are just as loyal to the United States as other Americans.

c. Compared to other groups, Jewish people have too much power in the media.

d. Jewish people talk about the Holocaust just to further their political agenda.

e. Jewish people chase money more than other people do.

1. Do not agree

2. Somewhat agree

3. Strongly agree

4. Very strongly agree

**Political violence**

**Q:** People have different views about violence in the United States. How much do you agree or disagree with each of the following statements?

a. If elected leaders will not protect American democracy, the people must do it themselves, even if it requires taking violent actions.^2^

b. Because things have gotten so far off track, true American patriots may have to resort to violence in order to save our country.^10^

c. Our American way of life is disappearing so fast that we may have to use force to save it.^2^

1. Do not agree

2. Somewhat agree

3. Strongly agree

4. Very strongly agree

**Q:** Some people talk about a second civil war in the United States. Which of the following comes closer to your view of what a second civil war might look like?

a. A second civil war would be like the first Civil War in the United States, with opposing armies and large battles.

OR

b. A second civil war would be like an insurgency or guerrilla war, with small groups attacking specific targets or people.

**Q:** How much do you agree or disagree with each of the following statements?

a. In the next few years, there will be civil war in the United States.

b. The United States needs a civil war to set things right.

1. Do not agree

2. Somewhat agree

3. Strongly agree

4. Very strongly agree

*Now we have a few questions about the use of force or violence. “Force or violence” means physical force strong enough that it could cause pain or injury to a person. A reminder: your responses will be kept confidential and anonymous*.

**Q:** People sometimes talk about using force or violence to achieve political objectives. In general, what do you think about using force or violence to advance an important political objective that you support—is it…?

1. Never justified

2. Sometimes justified

3. Usually justified

4. Always justified

**Q:** Your view of the use of force or violence to advance an important political objective might depend on the specific objective that was involved. What do you think about the use of force or violence in the following situations—is it never justified, sometimes justified, usually justified, or always justified?

a. To return Donald Trump to the presidency this year

b. To stop an election from being stolen

c. To stop people who do not share my beliefs from voting

d. To prevent discrimination based on race or ethnicity

e. To preserve an American way of life based on Western European traditions

f. To oppose the government when it does not share my beliefs

g. To oppose the government when it tries to take private land for public purposes

h. To protect the environment or stop climate change

i. To protect the rights of animals

1. Never justified

2. Sometimes justified

3. Usually justified

4. Always justified

**Q:** You said that in general, the use of force or violence was [response inserted] to advance an important political objective that you support. Your opinion might depend on the specific objective that was involved. What do you think about the use of force or violence in the following situations—is it never justified, sometimes justified, usually justified, or always justified?

a. To stop voter fraud

b. To stop voter intimidation

c. To stop police violence

d. To reinforce the police

e. To stop illegal immigration

f. To keep our borders open

g. To stop a protest or demonstration

h. To support a protest or demonstration

i. To preserve the American way of life l believe in

j. To oppose Americans who do not share my beliefs

1. Never justified

2. Sometimes justified

3. Usually justified

4. Always justified

*The next questions are about your personal willingness to use force or violence.*

(Questions asked of respondents who endorsed at least 1 use of violence to achieve a specific political objective.)

**Q:** In a situation where you think force or violence is justified to advance an important political objective, how willing would you personally be to use force or violence in each of these ways?

a. To damage property

b. To threaten or intimidate a person

c. To injure a person

d. To kill a person

1. Not willing

2. Somewhat willing

3. Very willing

4. Completely willing

(Question asked of all respondents.)

**Q:** Thinking now about the future and all the changes it might bring, how likely is it that you will use a gun in any of the following ways in the next few years—in a situation where you think force or violence is justified to advance an important political objective?

a. I will be armed with a gun.

b. I will carry a gun openly, so that people know I am armed.

c. I will threaten someone with a gun.

d. I will shoot someone with a gun.

1. Not likely

2. Somewhat likely

3. Very likely

4. Extremely likely

**ADDITIONAL METHODS TEXT**

The analysis was conducted by Bradley Velasquez with primary oversight by Garen Wintemute.

**Questionnaire design**

To minimize inattentive responses to questions regarding political violence, questions on that topic were immediately preceded by a question asking respondents about the justifiability of the use of force or violence in 7 non-political situations. These were presented to all respondents in a fixed order from what the authors considered likely to be seen as justifying violence (“in self-defense”) to unlikely (“to get respect”). This was done to create an expected response transition from support to nonsupport that respondents would need to reverse to indicate support for political violence.

**Model development**

We examined the following models for adjusting prevalence differences:

Model 0: unadjusted;

Model 1: adjusted for age (numerical), race and ethnicity (White, Non-Hispanic; Black, Non-Hispanic; Other, Non-Hispanic; Hispanic; 2+ Races, Non-Hispanic), and gender (Male, Female);

Model 2: additionally adjusted for income (Less than $10,000, $10,000 to $24,999, $25,000 to $49,999, $50,000 to $74,999, $75,000 to $99,999, $100,000 to $149,999, $150,000 or more), education (No high school diploma or GED, High school graduate (high school diploma or the equivalent GED), Some college or Associate's degree, Bachelor's degree, Master’s degree or higher), and Census division (New England, Mid-Atlantic, East-North Central, West-North Central, South Atlantic, East-South Central, West-South Central, Mountain, Pacific);

Model 3: additionally adjusted for rurality (Urban, Rural; derived from Rural-Urban Commuting Codes matched to census tracts (https://www.ers.usda.gov/data-products/rural-urban-commuting-area-codes/).

Model 3 was selected; findings from Model 3 appear in the ‘Adjusted prevalence difference” rows in tables. Q values for comparisons in the text were also produced using Model 3.

**ADDITIONAL RESULTS TEXT**

Seven items in the 2023 survey that were included in this analysis had nonresponse percentages above 3.0%.

**Q:** Some people talk about a second civil war in the United States. Which of the following comes closer to your view of what a second civil war might look like?

A second civil war would be like the first Civil War in the United States, with opposing armies and large battles.

OR

A second civil war would be like an insurgency or guerrilla war, with small groups attacking specific targets or people.

Nonresponse = 4.9%

**Q:** How much do you agree or disagree with each of the following statements about gender in the United States today?

I believe that the male/female dichotomy is natural.

Nonresponse = 5.4%

**Q:** How much do you agree or disagree with each of the following statements about people’s religious beliefs in the United States today?

Most Muslims living in the United States discriminate against women.

Nonresponse = 4.0%

Most Muslims living in the United States are hostile to the United States.

Nonresponse = 3.3%

Most Muslims living in the United States are more prone to violence than other people.

Nonresponse = 3.3%

Most Muslims living in the United States are less civilized than other people.

Nonresponse = 3.2%

**Q:** How much do you agree or disagree with each of the following statements about people in the United States today?

Having more Black Americans, Latinos, and Asian Americans is good for the country.

Nonresponse = 3.1%

**Table S1. Sociodemographic characteristics of respondents (weighted)**

This table previously appeared in Wintemute GJ, Robinson SL, Crawford A, et al. Single-year change in views of democracy and society and support for political violence in the USA: findings from a 2023 nationally representative survey. *Inj Epidemiol* 2024; **11**: 20.

**Table S2. Sociodemographic characteristics of respondents and non-respondents in the 2022 and 2023 surveys (unweighted)**

**Table S2, continued.**

Mean (SD) ages were as follows: Wave 1 responders, 55.7 (16.7); Wave 1 non-responders, 45.4 (16.8); Wave 1 respondents who left the panel prior to Wave 2, 52.17 (16.2); Wave 2 responders, 56.99 (16.5); Wave 2 non-responders, 52.47 (17.5).

This table previously appeared in the supplement to Wintemute GJ, Robinson SL, Crawford A, et al. Single-year change in views of democracy and society and support for political violence in the USA: findings from a 2023 nationally representative survey. *Inj Epidemiol* 2024; **11**: 20.

**Table S3. Prevalence of agreement with individual items used to construct phobia scales (n = 9385)**

**Table S3, continued.**

**Table S3, continued.**

**Table S4. Level of agreement with statements of homonegativity and beliefs regarding political violence** **(n = 9385)**

**Table S4, continued.**

**.**

See the methods section of the main manuscript for details of the categorization of respondents by level of agreement with homonegativity.

Prevalence differences are percentage point differences. They are weighted and adjusted for age, race and ethnicity, gender, education, income, Census division, and rurality. Response options for which these differences were calculated are as follows: for need for violence to effect social change and for civil war, strongly or very strongly agree; for violence to advance specific political objectives, “force or violence to advance an important political objective” was usually or always justified to advance at least 1 of 19 such objectives; for personal willingness to commit political violence, very or completely willing; for expectations of firearm use, very or extremely likely.

Q values represent the probability that the given difference would be a false discovery; they represent the expected proportion of “false positives” that would be seen among the collection of all differences whose q values were at or below the given q value.

**Table S5. Level of agreement with statements of racism and beliefs regarding political violence (n = 9385)**

**Table S5, continued.**

See the methods section of the main manuscript for details of the categorization of respondents by level of agreement with racism.

Prevalence differences are percentage point differences. They are weighted and adjusted for age, race and ethnicity, gender, education, income, Census division, and rurality. Response options for which these differences were calculated are as follows: for need for violence to effect social change and for civil war, strongly or very strongly agree; for violence to advance specific political objectives, “force or violence to advance an important political objective” was usually or always justified to advance at least 1 of 19 such objectives; for personal willingness to commit political violence, very or completely willing; for expectations of firearm use, very or extremely likely.

Q values represent the probability that the given difference would be a false discovery; they represent the expected proportion of “false positives” that would be seen among the collection of all differences whose q values were at or below the given q value.

**Table S6. Level of agreement with statements of transphobia and beliefs regarding political violence (n = 9385)**

**Table S6, continued.**

See the methods section of the main manuscript for details of the categorization of respondents by level of agreement with transphobia.

Prevalence differences are percentage point differences. They are weighted and adjusted for age, race and ethnicity, gender, education, income, Census division, and rurality. Response options for which these differences were calculated are as follows: for need for violence to effect social change and for civil war, strongly or very strongly agree; for violence to advance specific political objectives, “force or violence to advance an important political objective” was usually or always justified to advance at least 1 of 19 such objectives; for personal willingness to commit political violence, very or completely willing; for expectations of firearm use, very or extremely likely.

Q values represent the probability that the given difference would be a false discovery; they represent the expected proportion of “false positives” that would be seen among the collection of all differences whose q values were at or below the given q value.

**Table S7. Level of agreement with statements of** **xenophobia and beliefs regarding political violence (n = 9385)**

**Table S7, continued.**

See the methods section of the main manuscript for details of the categorization of respondents by level of agreement with xenophobia.

Prevalence differences are percentage point differences. They are weighted and adjusted for age, race and ethnicity, gender, education, income, Census division, and rurality. Response options for which these differences were calculated are as follows: for need for violence to effect social change and for civil war, strongly or very strongly agree; for violence to advance specific political objectives, “force or violence to advance an important political objective” was usually or always justified to advance at least 1 of 19 such objectives; for personal willingness to commit political violence, very or completely willing; for expectations of firearm use, very or extremely likely.

Q values represent the probability that the given difference would be a false discovery; they represent the expected proportion of “false positives” that would be seen among the collection of all differences whose q values were at or below the given q value.

**Table S8. Level of agreement with statements of** **hostile sexism and beliefs regarding political violence (n = 9385)**

**Table S8, continued.**

See the methods section of the main manuscript for details of the categorization of respondents by level of agreement with hostile sexism.

Prevalence differences are percentage point differences. They are weighted and adjusted for age, race and ethnicity, gender, education, income, Census division, and rurality. Response options for which these differences were calculated are as follows: for need for violence to effect social change and for civil war, strongly or very strongly agree; for violence to advance specific political objectives, “force or violence to advance an important political objective” was usually or always justified to advance at least 1 of 19 such objectives; for personal willingness to commit political violence, very or completely willing; for expectations of firearm use, very or extremely likely.

Q values represent the probability that the given difference would be a false discovery; they represent the expected proportion of “false positives” that would be seen among the collection of all differences whose q values were at or below the given q value.

**Table S9. Level of agreement with statements of Islamophobia and beliefs regarding political violence (n = 9385)**

**Table S9, continued.**

See the methods section of the main manuscript for details of the categorization of respondents by level of agreement with Islamophobia.

Prevalence differences are percentage point differences. They are weighted and adjusted for age, race and ethnicity, gender, education, income, Census division, and rurality. Response options for which these differences were calculated are as follows: for need for violence to effect social change and for civil war, strongly or very strongly agree; for violence to advance specific political objectives, “force or violence to advance an important political objective” was usually or always justified to advance at least 1 of 19 such objectives; for personal willingness to commit political violence, very or completely willing; for expectations of firearm use, very or extremely likely.

Q values represent the probability that the given difference would be a false discovery; they represent the expected proportion of “false positives” that would be seen among the collection of all differences whose q values were at or below the given q value.

**Table S10. Level of agreement with statements of antisemitism and beliefs regarding political violence (n = 9385)**

**Table S10, continued.**

See the methods section of the main manuscript for details of the categorization of respondents by level of agreement with antisemitism.

Prevalence differences are percentage point differences. They are weighted and adjusted for age, race and ethnicity, gender, education, income, Census division, and rurality. Response options for which these differences were calculated are as follows: for need for violence to effect social change and for civil war, strongly or very strongly agree; for violence to advance specific political objectives, “force or violence to advance an important political objective” was usually or always justified to advance at least 1 of 19 such objectives; for personal willingness to commit political violence, very or completely willing; for expectations of firearm use, very or extremely likely.

Q values represent the probability that the given difference would be a false discovery; they represent the expected proportion of “false positives” that would be seen among the collection of all differences whose q values were at or below the given q value.

**Table S11. Level of agreement with allophobia and beliefs regarding political violence (n = 9385)**

**Table S11, continued.**

See the methods section of the main manuscript for details of the categorization of respondents by level of agreement with allophobia (a combined measure).

Prevalence differences are percentage point differences. They are weighted and adjusted for age, race and ethnicity, gender, education, income, Census division, and rurality. Response options for which these differences were calculated are as follows: for need for violence to effect social change and for civil war, strongly or very strongly agree; for violence to advance specific political objectives, “force or violence to advance an important political objective” was usually or always justified to advance at least 1 of 19 such objectives; for personal willingness to commit political violence, very or completely willing; for expectations of firearm use, very or extremely likely.

Q values represent the probability that the given difference would be a false discovery; they represent the expected proportion of “false positives” that would be seen among the collection of all differences whose q values were at or below the given q value.

**REFERENCES**

1. Morrison MA, Morrison TG. Sexual orientation bias toward gay men and lesbian women: modern homonegative attitudes and their association with discriminatory behavioral intentions. *J Appl Soc Psychol* 2011; **41**(11): 2573–99.

2. Pew Research Center. Deep divisions in Americans’ views of nation’s racial history – and how to address it. 2021 August. https://www.pewresearch.org/politics/2021/08/12/deep-divisions-in-americans-views-of-nations-racial-history-and-how-to-address-it/.

3. Cox D, Lienesch R, Jones RP. Beyond economics: fears of cultural displacement pushed the white working class to Trump | PRRI/The Atlantic Report. Public Religion Research Institute. 2019 May 17. https://www.prri.org/research/white-working-class-attitudes-economy-trade-immigration-election-donald-trump/.

4. Pew Research Center. Americans see advantages and challenges in country’s growing racial and ethnic diversity. 2019 May. https://www.pewresearch.org/social-trends/2019/05/08/americans-see-advantages-and-challenges-in-countrys-growing-racial-and-ethnic-diversity/.

5. Nagoshi JL, Adams KA, Terrell HK, Hill ED, Brzuzy S, Nagoshi CT. Gender differences in correlates of homophobia and transphobia. *Sex Roles* 2008; **59**: 521–31.

6. Maloni MJ, Gligor DM, Blumentritt T, Gligor N. Fear or competition? Antecedents to U.S. business student immigration attitudes. *J Manag Educ* 2022; **46**(4): 715-50.

7. Glick P, Fiske ST. Hostile and benevolent sexism: measuring ambivalent sexist attitudes toward women. *Psychol Women Q* 1997; **21**:119–35.

8. Institute for Social Policy and Understanding. The National American Islamophobia Index. Undated. https://www.ispu.org/islamophobia-index/.

9. Allington D, Hirsch D, Katz L. The generalised antisemitism (GEAS) scale: validity and factor structure. *J Contemp Antisemitism* 2022; **5**(2): 1–28.

10. IFYC – PRRI Survey on Religion & COVID-19 Vaccine Trust. 2021 March. https://www.prri.org/wp-content/uploads/2021/05/Topline-IFYC-PRRI-Survey-on-Religion-and-COVID-19-Vaccine-Trust-v2_final.pdf.
